# Skeletal manifestations of Marfan syndrome associated to heterozygous R2726W *FBN1* variant: sibling case report and literature review

**DOI:** 10.1186/s12891-016-0935-9

**Published:** 2016-02-15

**Authors:** Octavio D. Reyes-Hernández, Carmen Palacios-Reyes, Sonia Chávez-Ocaña, Enoc M. Cortés-Malagón, Patricia Garcia Alonso-Themann, Víctor Ramos-Cano, Julián Ramírez-Bello, Mónica Sierra-Martínez

**Affiliations:** Laboratorio de Genética y Diagnóstico Molecular, Hospital Juárez de México, Instituto Politécnico Nacional 5160, Gustavo A. Madero, Magdalena de Las Salinas, Ciudad de México, DF 07760 Mexico; Seguimiento Pediátrico, Instituto Nacional de Perinatología, Montes Urales 800 Col. Lomas de Chapultepec, Del. Miguel Hidalgo, 11000 Ciudad de México, Mexico; Servicio de Cirugía Cardio-torácica, Hospital Juárez de México. Av, Instituto Politécnico Nacional 5160, Gustavo A. Madero, Magdalena de Las Salinas, 07760 Ciudad de México, Mexico; Laboratorio de Medicina Genómica, Hospital Juárez de México, Instituto Politécnico Nacional 5160, Gustavo A. Madero, Magdalena de Las Salinas, 07760 Ciudad de México, Mexico

**Keywords:** *FBN1* gene, R2726W variant, Type-1 fibrillinopathy

## Abstract

**Background:**

*FBN1* (15q21.1) encodes fibrillin-1, a large glycoprotein which is a major component of microfibrils that are widely distributed in structural elements of elastic and non-elastic tissues. *FBN1* variants are responsible for the related connective tissue disorders, grouped under the generic term of type-1 fibrillinopathies, which include Marfan syndrome (MFS), MASS syndrome (Mitral valve prolapse, Aortic enlargement, Skin and Skeletal findings, Acromicric dysplasia, Familial ectopia lentis, Geleophysic dysplasia 2, Stiff skin syndrome, and dominant Weill-Marchesani syndrome.

**Case presentation:**

Two siblings presented with isolated skeletal manifestations of MFS, including severe pectus excavatum, elongated face, scoliosis in one case, and absence of other clinical features according to Ghent criteria diagnosis, were screened for detection of variants in whole *FBN1* gene (65 exons). Both individuals were heterozygous for the R2726W variant. This variant has been previously reported in association with some skeletal features of Marfan syndrome in the absence of both tall stature and non-skeletal features. These features are consistent with the presentation of the siblings reported here.

**Conclusion:**

The presented cases confirm that the R2726W *FBN1* variant is associated with skeletal features of MFS in the absence of cardiac or ocular findings. These findings confirm that *FBN1* variants are associated with a broad phenotypic spectrum and the value of sequencing in atypical cases.

## Background

*FBN1* is located in chromosome 15q21.1, comprising approximately 200 kb of genomic DNA, and is made up of 65 exons [1, 2]. *FBN1* encodes fibrillin-1, a large glycoprotein with a molecular weight of approximately 350 kDa [3], a major component of 10–12 nm connective tissue microfibrils that are widely distributed in both elastic and non-elastic tissues; it is assembled into extracellular matrix microfibrils, which are structural components of connective tissues [2, 3]. Mutations in the fibrillin-1 gene (*FBN1*; MIM 134797) are responsible for disorders of the connective tissue, grouped under the generic term of type-1 fibrillinopathies which include Marfan syndrome (MFS/MIM 154700), MASS syndrome [Mitral valve prolapse, Aortic enlargement, Skin and Skeletal findings (MIM 604308)], familial ectopia lentis (MIM 129600), geleophysic dysplasia 2 (MIM 614185), stiff skin syndrome (MIM 184900), acromicric dysplasia (MIM 102370) and dominant Weill-Marchesani syndrome 2 (MIM 608328) [4–8]. The first described and most frequently reported condition associated with *FBN1* mutations is MFS, an autosomal dominant inherited disorder of the connective tissue with a prevalence of 1 in 5,000 affected individuals [5].

Marfan syndrome affects the skeletal, ocular, and cardiovascular systems, as well as the skin, lungs and dura. There is tremendous phenotypic variability in terms of age of presentation (particularly in terms of cardiac and ocular findings), tissues affected and severity of manifestations between and within families. The presence of these clinical features in conjunction and the autosomal dominant inheritance pattern are suggestive of a MFS diagnosis. Due to the clinical variability, diagnostic criteria have been defined for patients with or without a positive family history of MFS [9, 10]. Revised Ghent criteria for MFS diagnosis, establishes four scenarios in absence of family history for a proband: Aortic root enlargement (Z-score ≥2.0) plus ectopia lentis, or with a pathogenic *FBN1* variant; or with a systemic score ≥7; or with ectopia lentis and *FBN1* pathogenic variant. In the presence of familial cases (a first-degree relative of the proband affected with MFS), diagnosis can be established with the presence of ectopia lentis or aortic root enlargement (Z-score ≥2.0 in those age ≥20 years or ≥3.0 in those age <20 years) or systemic score ≥7. Scoring of systemic features include the presence of thumb and wrist sign (3 points), either wrist or thumb sign (1), pectus carinatum (2), hind foot deformity (2), pneumothorax (2), dural ectasia (2), protrusio acetabuli (2), reduced upper segment/lower segment and increased arm/length ratio and no severe scoliosis (1), scoliosis or thoracolumbar kyphosis (1), reduced elbow extension (1), 3/5 facial features like dolichocephaly, enophtalmos, downslanting palpebral fissures, malar hypoplasia and retrognathia (1), skin striae (1), myopia > 3 diopters (1), mitral valve prolapse (1), pectus excavatum or chest asymmetry (1) and plain pes planus (1). It is clear that these criteria emphasis the cardiovascular and ocular manifestations as the cardinal clinical features, and focuss less on the musculoskeletal signs to systemic scores, which were more important in the original Ghent criteria. In fact, from 16 points evaluated in the score system, 11 correspond to skeletal manifestations [10, 11].

The vast majority of individuals meeting diagnostic criteria for MFS have mutations in FNB1 [12, 13]. However, *FBN1* variants may also be present in individuals with incomplete Ghent criteria (without complete phenotype or with isolated findings) [13, 14]; i.e. inherited forms affecting only one of the systems involved in MFS, like isolated ectopia lentis, isolated ascending aortic aneurysm and/or dissection (AAD) or skeletal manifestations [14–16]. In MFS, besides skeletal manifestations defined in the score system, other features including but not limited to tall stature, arachnodactyly, long slender limbs, are frequently observed [11, 17]. Interestingly, some families have been found to carry *FBN1* gene variants associated with isolated Marfan-like skeletal features [18–20].

In MFS and related fibrillinopathies, the structural and functional complexity of microfibrillin formed mainly by fibrillin-1 is affected since the microfibrillin plays a role in tropoelastin deposition and elastic fiber formation, in addition to possessing an anchoring function in some tissues. Several *FBN1* variants result in the production of abnormal fibrillin-1 proteins that, when incorporated into microfibrils along normal fibrillin proteins, cause a structurally inferior connective tissue [21, 22]. Among the *FBN1* mutations implicated in functional changes or disease, a cluster located in exons 59 to 65 is associated with the mild phenotype, typified by a lack of significant aortic pathology [21]. Three mutually non-exclusive models of the pathophysiology of MFS have been proposed: a dominant negative model, disturbance of tissue homeostasis, and increased susceptibility of fibrillin to proteolysis [7, 23]. Although genotype–phenotype correlations have been attempted, few potential ones have emerged, and the underlying molecular correlates remain unclear [12–14].

In this paper we present the clinical findings of two siblings from a Mexican family with only skeletal manifestations of MFS and screening analysis of the *FBN1* gene variants [24]. Our patients are compared with the previously reported clinical features of the same variant, in order to determine the most frequent characteristics associated to *FBN1* R2726W variant [18–20]. We also describe which features led us to classify these cases as a type-1 fibrillinopathy related to Marfan-like skeletal manifestations.

## Case presentation

**Fig. 1:**
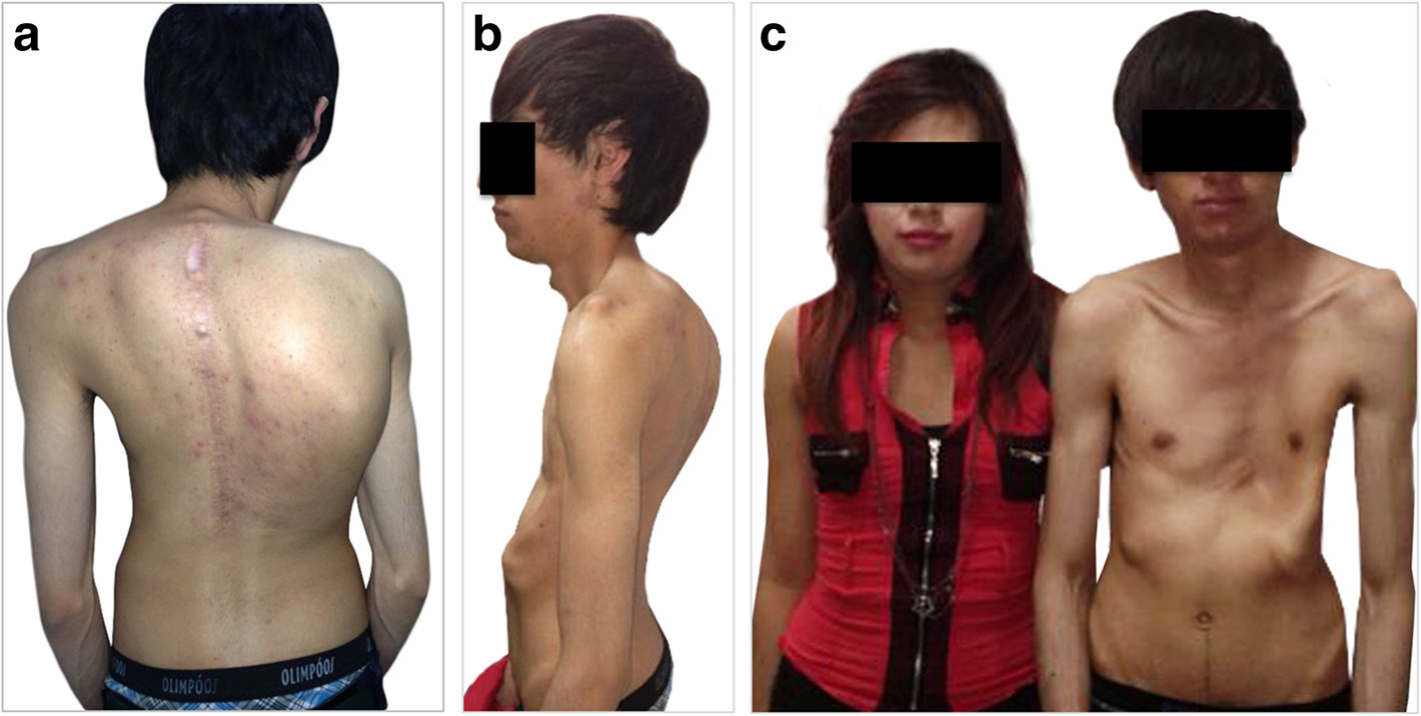
Clinical features of the proband and his sister. **a**, **b** Clinical phenotype of the proband (II:2) including severe pectus excavatum, scoliosis and some Marfan facial features. **c** Clinical phenotypes of the proband and his sister (II:3) including facial features

**Fig. 2:**
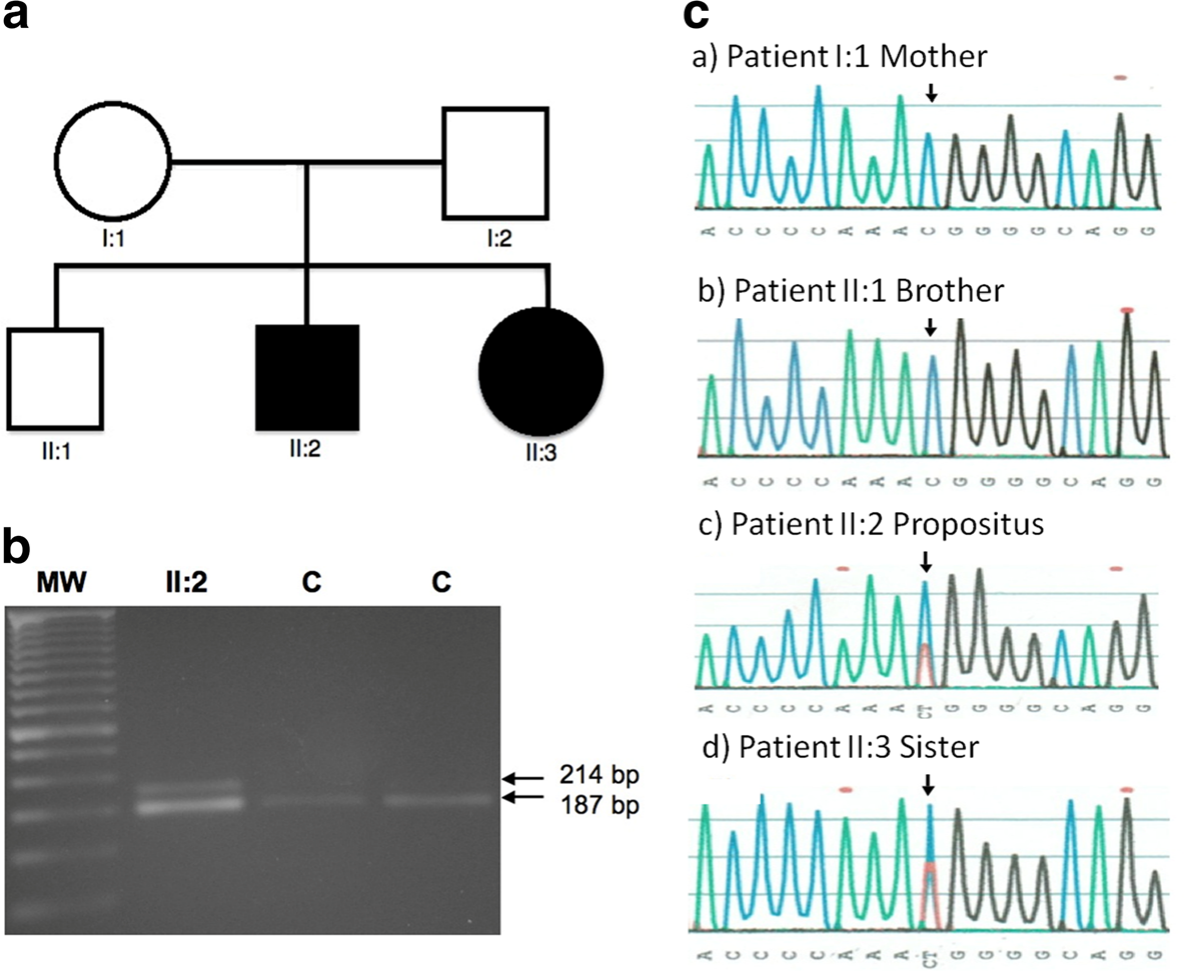
Family pedigree and molecular characterization of the *FBN1* variant. **a** Family pedigree. *Black square* and *circle* indicates the individuals affected (II:2, II:3). **b** PCR-RFLP analysis of the R2726W *FBN1* variant. The 214-bp amplification product was submitted to MspI cleavage. Lines 2 and 3, heterozygous variant (patients II:2 and II:3); lines 3 and 4, wild-type genotype (I:2 and II:1). MspI cleaves a 214-bp fragment into 27- and 187-bp fragments in the presence of the C wild-type allele, whereas the T mutant allele is not cleaved. **c** The R2726W variant in exon 64 of *FBN1.* Electropherogram showing the corresponding normal sequence in unaffected family members: *a*) Mother (I:1), *b*) brother (II:1), *c* and *d*) the C to T heterozygous variant in II:2 and II:3, indicated by the *arrow*, resulting in the substitution of arginine by tryptophan (R2726W)

Patient 1. The proband was an 18 year old boy (Figs. 1a, b, c and 2a (individual II:2)) who at the time of the first evaluation was 16. On examination we found severe pectus excavatum, scoliosis and a Marfan facial appearance (malar hypoplasia, enophthalmos, retrognathia), as well as elongated face, high arched palate and crowded teeth. The proportion of segments was normal and he was of normal height (25^th^–50^th^ centile), had no cardiac symptoms He has remained asymptomatic with no chest pain, syncope, or heart murmurs. No abnormalities were detected on electrocardiograma or echocardiogram. Ocular examination was normal, specifically lens placement was normal and there was no evidence of myopia. Computed Tomography (CT) imaging found there was no evidence of dural ectasia or protusio acetabulae. Annual evaluations since that time have failed to detect any cardiovascular, ocular or systemic involvement. The proband did not fulfill criteria for MFS or related disorders, since he presented one major and two minor criteria of the skeletal system according to Ghent criteria, with a total of 3 points for the score in the revised Ghent Criteria10–11. We then evaluated the rest of the family members.

Patient 2. The proband’s sister (Figs. 1c and 2a (Individual II:3)), a 16 year old girl was diagnosed with moderate pectus excavatum at 14 years of age. She also had some Marfanoid facial features (enophthalmos, retrognathia and malar hypoplasia). Height was normal (50th–75th centile). She currently has no cardiovascular or ocular abnormalities. According to Ghent criteria the patient had two minor skeletal criteria and 2 points for the revised score.

Clinical examination of the proband’s mother (Fig. 2a, individual I:2) and brother (Fig. 2a, individual II:1) did not show any skeletal or marfanoid features. Unfortunately the father (Fig. 2a, individual I:1) was not available for clinical evaluation or sample collection.

### Mutational screening

Genomic DNA was isolated from peripheral blood lymphocytes using the QIA amp DNA blood mini kit (Qiagen, Inc) according to the manufacturer´s instructions. All 65 exons of *FBN1* were PCR amplified using specific primers as described previously [8], and sequenced directly using a DNA Sequencing Kit with Big Dye Terminator on an automated ABI PRISM 3100 DNA sequencer (Applied Biosystems, Foster City, CA, USA). To detect the R2726W *FBN1* variant by PCR-RFLP, a 214-bp fragment was amplified by PCR using the following primers: 5'-GAGTGTAAGATCAATGGCTACCCCAAC-3' (forward) and 5'-CTAGAGCTCAGGGAATGTCTGGAGTTTC-3' (reverse). Reaction parameters included 35 cycles of melting at 94 °C for 30 s, annealing at 60 °C for 20 s, and extension at 72 °C for 25 s, followed by a 5 min final extension at 72 °C. The forward primer contained an artificial cytosine at the 3' end, which generates an MspI endonuclease recognition site. The R2726W (C to T transition at nucleotide 8,176) substitution eliminates the MspI endonuclease recognition site (Fig. 2). The 214-bp amplification product was submitted to MspI cleavage, and the resultant fragments were electrophoresed in a 2 % agarose gel containing ethidium bromide.

### Molecular analyses

Mutational screening analysis in the proband revealed the heterozygous c.C8176T transition in exon 64 of the *FBN1* gene, corresponding to R2726W variant in protein FBN1, which leads to a single amino acid substitution of tryptophan for arginine. His sister (Fig. 2a, individual II:3), is heterozygous for the same variant. The mother and one brother (Fig. 2a, individuals I:2 and II:1, respectively) did not carry the R2726W variant [Fig. 2c]. PCR-RFLP analysis was used to confirm the R2726W *FBN1* variant found by direct sequencing in the proband II:2 (Fig. 2b). Unfortunately, it was not possible to test the father, but probably the variant is paternally inherited.

## Discussion

Several pathogenic *FBN1* variants have been identified in MFS, affecting mostly the skeletal, ocular and cardiovascular systems [25–32]. However, there are families affected with only one of these systems, carrying *FBN1* inherited variants [27–30] (i.e. ascending aortic aneurysm and dissection [27, 28], isolated ectopia lentis [28] and isolated skeletal features [18–20]). The siblings presented here had isolated skeletal features and were found to be heterozygous for the R2726W *FBN1* variant (rs61746008) in exon 64, which has been previously reported in similarly affected individuals. It encodes a region in the single carboxy terminal domain of fibrillin that contains a recognition motif for proteolytic tetrabasic cleavage, which undergoes extracellular processing by an enzyme of the PACE (Paired basic Amino acid Cleaving Enzyme) convertase family. Furin/PACE specifically acts at the carboxy terminal cleavage site of profibrillin-1 to convert it into fibrillin [33, 34]. R2726W variant affects the processing of profibrillin into fibrillin, and fibroblasts harbouring this variant reduce fibrillin deposition in vivo, which results in a lack of its incorporation into microfibrils [18]. Up to now, families with 16 individuals affected by this variant have been reported, showing variability in clinical manifestations: some individuals manifested tall stature, others skeletal abnormalities and others had no clinical signs [18–20, 30–32].

The R2726W *FBN1* variant was first described in 1995, in a 13 year old patient with tall stature, pectus carinatum, scoliosis, arachnodactyly and pes planus, leading to major involvement of the skeletal system. Five additional members of the family with the variant presented only with tall stature, including one with metacarpal index above the mean [18]. Additionally, in 2004, the R2726W *FBN1* variant was reported in two individuals with average height from one family [19]. The son (proband) inherited the maternal variant and had a Marfan-like phenotype, with skeletal, ocular and cardiac systems affected. Skeletal manifestations were characterized by marfanoid habitus and facial characteristics (dolichocephaly, malar hypoplasia, retrognathia, down-slanting palpebral fissures and highly arched palate), pectus carinatum and medial displacement of the medial malleolus causing pes planus. Cardiac and ocular features were also present in the form of prolapse of the leaflet of the tricuspid valve, mild myopia and alternating exotropia. He also presented with unrelated conditions including aggressive and hyperactive behavior, a slight increase in the size of the cerebral ventricles and the vermian cistern, developmental delay, childhood febrile and non-febrile seizures. The features in the proband were suggestive of Lujan–Fryns syndrome. The mother had a history of treated childhood seizures. It was proposed that variant was associated with incomplete penetrance.

In 2008, a female with mitral valve prolapse, myopia and skin striae was reported [32]. In 2009, a family of four affected individuals was described with two individuals affected who were heterozygous for the R2726W variant, which was also restricted to skeletal features of MFS. The third affected family member was heterozygous for a C1928S variant and the last one was compound heterozygous. Both satisfied the Ghent criteria, probably due to the C1928S variant. The sibling who was heterozygous for the R2726W *FBN1* variant presented the following clinical manifestations: mild scoliosis, articular hypermobility, pes planus, highly arched palate, and mild pectus excavatum. The proband’s father, who also was heterozygous, had mild pectus excavatum, wrist and thumb signs [20]. In 2013, three members of a family were reported where the proband had skeletal features of MFS in conjunction with several birth defects, epilepsy, language and behavior alterations attributable to the 17q21.31 microdeletion. The father and sister who were also heterozygous for the variant showed some skeletal features of MFS [31]. Finally, in 2014, another individual with MFS criteria was reported, with a compound heterozygous genotype [30].

On Tables 1 and 2, we compared the skeletal characteristics of all the heterozygous individuals previously reported (13 cases), according to Ghent Criteria and the Revised Ghent Criteria respectively. The most frequent features are tall stature (4/13), pectus alterations (4/13), facial appearance (3/10), kypho-scoliosis (3/13) and pes planus (2/10). Only 3 had ocular features (myopia) and 2 cardiovascular features (tricuspid and mitral valve prolapse). In this report, both siblings carrying the heterozygous R2726W *FBN1* variant are of average height. The 18 year old brother had a more severe phenotype compared to the 16-year-old sister. They present clinical features similar to the heterozygous son and father reported by Dikj in 2004. The son was 17 years and presented with kyphosis, both he and his father had pectus deformities [22]. Including the siblings presented here to previously reported cases, the most frequent clinical features were pectus deformity (6/15), facial features (5/15), tall stature (4/15) and kypho-scoliosis (4/15). Therefore, this variant is clearly involved with skeletal manifestations of MFS. Although it was not possible to test the father, it is likely that the variant was paternally inherited.

**Table 1:** Clinical skeletal features of individuals affected with R2726W FBN1 mutation according to Ghent criteria

	Individuals/Case number																	
Case number	1^a^	2^a^	3^a^	4^a^	5^a^	6^a^	7^b^	8^b^	9^c^	10^d^	11^d^	12^d^	13^e^	14^e^	15^e^	16^f^	17^g^	18^g^
Sex	f	m	f	m	f	m	m	f	F	m	m	m	m	f	m	m	m	f
Age	67	37	46	22	17	13	18	41	20	54	17	20	ND	ND	21	19	18	16
FBN1 Variant	R2726W	R2726W	R2726W	R2726W	R2726W	R2726W	R2726W	R2726W	R2726W	R2726W	R2726W	R2726W/C1928S	R2726W	R2726W	R2726W/17q21.31 microdel	R2726W/R636Gly	R2726W	R2726W
Heigh (cm/TS)	169/-	193/+	173/-	191/+	175.2/+	183/+	178/-	164	ND/-	ND/-	ND/-	ND/-	ND/-	ND/-	168/-	ND/-	175/-	166/-
**Skeletal features**																		
**Major**																		-
Pectus carinatum	-	-	-	-	+	+	-	-		-	-	-	-	-	-	-	-	-
Pectus excavatum requiring surgery	-	-	-	-	-	-	-	-		-	-	+	-	-	-	-	-	-
Arm span to height ratio >1.05	-	-	-	-	-	-	-	-		-	-	+	-	-	-	-	-	-
Wrist and thumb signs	-	-	-	-	-	-	-	-		+	-	+	-	-	-	-	-	-
Scoliosis or spondylolisthesis	-	-	-	-	-	-	-	-		-	-	+	-	+	+	-	+	-
Reduced extension at the elbows	-	-	-	-	-	-	-	-		-	-	-	-	-	-	-	-	-
Pes planus	-	-	-	-	-	+	-	-		-	+	+	-	-	-	-	-	-
Protrusio acetabulae of any degree (RX)	-	-	-	-	-	-	-	-		ND	ND	ND	-	-	-	-	-	-
**Minor**																	-	-
Pectus excavatum of moderate severity	-	-	-	-	-	-	-	-		+	+	-	-	-	-	-	+	+
Joint hypermobility	-	-	-	-	-	+	-	-		-	+	+	-	-	-	-	-	-
High arched palate with crowding of teeth	-	-	-	-	-	HAP	-	-		-	-	+	+	HAP	CT	-	-	-
Facial appearance	-	-	-	-	-	-	+	-	-	-	-	**+**	**-**	-		**-**	+	+
Cardiovascular features	-	-	-	-	-	-	TVP	-	MVP	-	-	AoDil, AoDis	-		-	AI, BAV, MVP	-	-
Ocular features	-	-	-	-	-	-	My, Ex	-	My	-	-		My	-	My	My	-	**-**
Additional features	-	-	-	MI	-	-	AR, BA, ID, Ep C, BD	Ep C	St	-	HA	HA, St	Do, Ar	Do, St	Do, Ar, Ep,	-	En, MH, R, HAP, CT	En, MH, R

Individuals reported by: ^a^Milewicz et al. 1995 [18]; ^b^Buoni et al. 2004 [19]; ^c^Attanasio et al. 2008 [32]; ^d^Van Dijk 2009 [20]; ^e^Callier et al. 2013 [31]; ^f^Pepe et al. 2014 [30]; ^g^This report

A plus sign (+) denotes feature present, a minus sign (−) denotes feature absent

Other abbreviations:

*F* female, *m* male

*ND* information not described

*TS* Tall stature

*US/LS* Upper segment/Lower segment

*Microdel* microdeletion

Facial features: *Do* Dolichocephaly, *En* Enophthalmos, *DPF* Downslanting palpebral fissures, *MH* Malar hypoplasia, *R* Retrognatia, *HAP* High arched palate, *CT* Crowded teeth, *TVP* Tricuspide valve prolapse, *MPV* Mitral prolapse valve, *AoDil* Aorta dilatation, *AoDis* Aortic disection, *BAV* Bivalva aortic valve, *AI* Aortic insufficiency

*My* Myopia, *Ex* Exotropia

*MI* Metacarpal index above the mean

*BA* Behavior alterations

*ID* Intellectual disability

*Ep C* Epilepsy in childhood

*BD* Brain defects

*Ar* Aracnodactyly

**Table 2:** Clinical features of individuals affected with R2726W *FBN1* variant, according to the Revised Marfan criteria

	Individuals/Case number																		
Case number	Score	1^a^	2^a^	3^a^	4^a^	5^a^	6^a^	7^b^	8^b^	9^c^	10^d^	11^d^	12^d^	13^e^	14^e^	15^e^	16^f^	17^g^	18^g^
Sex		f	m	f	m	f	m	m	f	F	m	m	m	m	f	m	m	m	f
Age		67	37	46	22	17	13	18	41	20	54	17	20	ND	ND	21	19	18	16
FBN1 Variant		R2726W	R2726W	R2726W	R2726W	R2726W	R2726W	R2726W	R2726W	R2726W	R2726W	R2726W	R2726W/C1928S	R2726W	R2726W	R2726W/17q21.31 microdel	R2726W/R636Gly	R2726W	R2726W
Heigh (cm/TS)		169/-	193/+	173/-	191/+	175.2/+	183/+	178/-	164	ND/-	ND/-	ND/-	ND/-	ND/-	ND/-	168/-	ND/-	175/-	166
Skeletal eatures Score System																			
Arm span to height ratio	1	−	−	−	−	−	−	−	−	−	−	−	+	−	−	−	−	−	−
Wrist and thumb sign	3	−	−	−	−	−	−	−	−	−	+	+	+	−	−	−	−	−	−
Wrist or thumb sign	1	−	−	−	−	−	−	−	−	−	−	−	−	−	−	+	−	−	−
Pectus carinatum deformity	2	+	−	−	−	−	−	+	−	−	−	−	−	−	−	−	−	−	−
Pectus excavatum or chest assymetry	1	−	−	−	−	−	−	−	−	−	+	+	+	−	−	−	−	+	+
Hindfoot deformity	2	−	−	−	−	−	−	+	−	−	−	−	+	−	−	−	−	−	−
Pes planus	1	+	−	−	−	−	−	−	−	−	−	+	+	−	−	−	−	−	−
Protrusio acetabuli	2	−	−	−	−	−	−	−	−	−	−	−	−			−	−	−	−
Reduced US/LS and increased arm/height and no severe scoliosis	1	−	−	−	−	−	−	−	−	−	−	−	−	−	−	−	−	−	−
Scoliosis or thoracolumbar kyphosis	1	+	−	−	−	−	−	−	−	−	−	+	−	−	+	CT	−	+	−
Reduced elbow extension	1	−	−	−	−	−	−	−	−	−	−	−	−	−	HAP	−	−	−	−
Facial features (3/5)	1	+	−	−	−	−	−	+	−	−	−	−	+	−	−	−	−	+	+
Cardiovascular features		**-**	**-**	**-**	**-**	**-**	**-**	TVP	−	MVP	−	−	AoDil, AoDis	−		−	AI, BAV, MVP	−	−
Ocular features		−	−	−	−	−	−	My, Ex	−	My	−	−		My	−	My	My	−	−
Additional features		−	−	−	MI	−	−	AR, BA, ID, Ep C, BD	Ep C	St	−	HA	HA, St	Do, Ar	Do, St	Do, HA, Ar, Ep,	−	En, MH, R, HAP, CT	En, MH, R

Individuals reported by: ^a^Milewicz et al. 1995 [18]; ^b^Buoni et al. 2004 [19]; ^c^Attanasio et al. 2008 [32]; ^d^Van Dijk 2009 [20]; ^f^Pepe 2014 [30]; ^g^This report

A plus sign (+) denotes feature present, a minus sign (−) denotes feature absent

Other abbreviations:

*F* female, *m* male

*ND* information not described

*TS* Tall stature

*US/LS* Upper segment/Lower segment

*Microdel* microdeletion

Facial features: *Do* Dolichocephaly, *En* Enophthalmos, *DPF* Downslanting palpebral fissures, *MH* Malar hypoplasia, *R* Retrognatia, *HAP* High arched palate, *CT* Crowded teeth, *TVP* Tricuspide valve prolapse, *MPV* Mitral prolapse valve, *AoDil* Aorta dilatation, *AoDis* Aortic disection, *BAV* Bivalva aortic valve, *AI* Aortic insufficiency

*My* Myopia, *Ex* Exotropia

*MI* Metacarpal index above the mean

*NA* Not applicable

*BA* Behavior alterations

*ID* Intellectual disability

*Ep C* Epilepsy in childhood

*BD* Brain defects

*HA* Hyperlaxitud articular

*Ar* Aracnodactyly

Tables 1 and 2 clearly show that affected individuals with this variant have clinical variability ranging from no features (4/13) to isolated Marfan-like skeletal features (9/13) and incomplete penetrance. This is consistent with Bouni’s observations about R2726W variant, which is not necessarily related to tall stature or Marfanoid habitus. The variable expressivity is similar to that observed in MFS, since 12–21 % of individuals with *FBN1* variants had incomplete MFS clinical criteria [9, 10].

The phenotypic diversity is also consistent with the Universal Mutation Database [7], where to date over 1850 different *FBN1* pathogenic variants related to MFS and its associated disorders have been registered and the recorded R2716W variant was reported in conjunction with no clinical features, skeletal manifestations or other features. Looking for pathogenicity of some genetic variants previously associated with Marfan syndrome, population studies by next-generation sequencing found a prevalence of 0.002 for R2627W variant in European Americans and African Americans individuals [35]. The frequency is similar in the ExAc database [36], with an allelic frequency record of 0.00233 in Latin population and an overall frequency of 0.00073 including different populations (in 60706 unrelated individuals), and 1 heterozygous individual from America in 1000 genomes database from 26 different populations [37]. The low frequency of the variant does not exclude it from causing disease, and instead makes it a more credible cause.

Against the R2726W *FBN1* variant potential pathogenicity, are the heterozygous individuals without manifestations in described families, the presence of the variant in compound heterozygous state and one homozygous individual reported in ExAC database. However, there is no clinical description for the homozygous individual in ExAC database, since only individuals affected by severe pediatric disease have been removed. Variable expressivity and penetrance could rely on other factors. In vitro experiments have demonstrated that the R2726W *FBN1* variant affects the cleavage mediated by Furin/PACE and that profibrillin is unable to convert into fibrillin [33]. Of note, an in vitro study of R2726W/wild-type *FBN1* cells from a patient, had only half the amount of normal fibrillin incorporated into the extracellular matrix, resembling the effect of a non-expressed or “null” *FBN1* allele [18]. However, the features ranging from no phenotype to isolated skeletal manifestation could be explained by genetic modifiers of the wild-type *FBN1* allele implying a role for haploinsufficiency in MFS, since the tetrabasic consensus sequence is not absolutely required for C-terminal processing of fibrillin-1. C-terminal processing is not needed for fibrillin-1 function or utilization; modifiers are subject to tissue-specific modifications [33, 35].

However, since *FBN1* polymorphisms had been associated with tall stature, and isolated scoliosis in non-Marfan patients, clinical variability may be implied by allelic variants in *FBN1* expression or by certain *FBN1* polymorphisms [38]. Alternatively, modifier genes, such as *TGF-β* and *FBN2*, or even polymorphisms located in the *FBN1* gene may influence the penetrance or severity of MFS [39]. Therefore, involved mechanisms affecting the R2627W *FBN1* variant expression could be similar, and its penetrance may rely on the presence of other variants in *FBN1* or other genes involved in the same pathway as suggested by Van Dijk et al. [20].

Skeletal manifestations have also been associated to other variants. R1170H *FBN1* variant has been reported in a family with dolichostenomelia and arachnodactily [40] and G1796E in a family with kyphoscoliosis and radiological abnormalities of the spine, an unusual autosomal dominant condition with variable and progressive severity, without ocular or cardiac manifestations of MFS [41].

Although the number of reported cases of R2726W variant is rare, we think the frequency of such phenotypes may be underestimated. Reported cases to date point to skeletal manifestations like kypho-scoliosis and pectus deformities, as the most frequent features associated with the variant R2726W, therefore, families with this features should be screened for this variants regardless of the presence or absence of tall stature or Marfanoid habitus.

It is important to note that there was no follow up data available on any of the reported cases and, thus is possible that some individuals subsequently developed other MFS manifestations. Additional data needs to be gathered on other individuals carryng the data including follow up evaluations in order to better characterize the phenotype associated with this genotype. Since MFS phenotype appears in the first two decades of life, and since some of them have cardiovascular and ocular manifestations, it would be important to monitor carriers and to determine if they have cardiac, ocular or other system involvement, or if they complete clinical criteria for MFS. If they continue without changes, we could consider a R2627W *FBN1* variant related to Marfan-like skeletal manifestations. Also, in agreement with Faivre, a patient with isolated skeletal phenotype and R2726W variant could be classified as having type I fibrillinopathy. Nonetheless, given the significant phenotypic variability between and within families, wether attributable to compound heterozygosity or the presence of other possible modifiers in *FBN1* and related genes, cautious ongoing surveillance is always prudent.

## Conclusion

We confirmed the R2726W *FBN1* variant in siblings with isolated Marfan-like skeletal features. All clinical features for the reported cases support the expansion of the type-1 fibrillin related disorders, and reinforce the importance of skeletal findings in clinical practice in order to identify individuals with a possible fibrillinopathy. Findings in these siblings still show the clinical variability observed for this variant, and denote the *FBN1* sequence screening utility. Certainly, these cases represent a diagnostic challenge, and for individuals with this kind of skeletal abnormalities, the molecular mutation detection is necessary to establish the final diagnosis. The R2726W variant identification can be a valuable diagnostic aid in uncertain cases. Variant identification for the siblings we reported was helpful for genetic counseling, and appropriate follow up.

## Consent

The local Research Ethics Committee of Hospital Juarez de Mexico approved in accordance with the Declaration of Helsinki Principles, the clinical and genetic program for this study, and written informed consent was obtained from each individual and their parents before sample collection for genetic analyses. The four family members underwent complete clinical examination by the involved area specialists. Written informed consent was obtained from each patient and their parents for the publication of this case report and any accompanying images.

## Abbreviations

CT: computed tomography
MFS: Marfan syndrome
